# Predicting heart transplant outcomes using explainable artificial intelligence: a multicenter study

**DOI:** 10.3389/fcvm.2026.1649316

**Published:** 2026-03-13

**Authors:** Xin Zhou, Huangtao Sun, Aimin Xie, Zhihong Qiu, Guansen You, Dongmei Yang

**Affiliations:** Jiangxi Provincial People’s Hospital, The First Affiliated Hospital of Nanchang Medical College, Nanchang, China

**Keywords:** artificial intelligence, deep learning, heart transplantation, interpretable machine learning, postoperative prognosis

## Abstract

Due to the limited availability of donor hearts, precise and transparent prediction of post-transplant outcomes is critical for optimizing recipient selection and ensuring long-term survival. In this study, we propose a novel machine learning framework named the Generalizable Interpretable Neural Network (GINN), designed to achieve both high predictive accuracy and full model interpretability for survival prognosis following heart transplantation. GINN operates on structured clinical features using an additive representation approach, enabling explicit attribution of risk contributions from each clinical factor. We developed the GINN model based on comprehensive heart transplant data to predict one-year mortality and externally validated it on four independent cohorts. Using the same development data for training, the GINN model demonstrated robust predictive performance across three large-scale international transplant databases: United Network for Organ Sharing (UNOS 1994–2024, n=144,979), Eurotransplant registry (n=3,061) and Scandiatransplant registry (1997–2018, n=1,546), achieving AUROC scores of 0.827, 0.789, and 0.776 respectively. These results indicate strong generalizability and cross-population transferability. Moreover, in a small dataset from the Department of Cardiac Surgery, Jiangxi Provincial People's Hospital (External-CN, n=14), GINN maintained high risk identification capability with an AUROC of 0.821. The model constructed risk response functions based on nine key clinical variables, elucidating the marginal effects of donor and recipient age, donor function, preoperative support measures, and diagnostic types on postoperative risk. The findings suggest that GINN offers excellent generalization across geographic and sample-scale domains while maintaining predictive accuracy and providing stable and traceable risk explanations on structured clinical tabular data.

## 1 Introduction

Heart transplantation remains the most effective treatment for patients with end-stage heart failure and other advanced cardiac diseases. However, with global population ageing and the rising incidence of cardiovascular disorders, the demand for donor hearts now far outstrips supply (1–3). This discrepancy exposes many wait-listed patients to substantial mortality risk. Even after transplantation, recipients must contend with immune rejection, infection and drug-related complications, underscoring the need for accurate risk assessment and survival prediction.

To optimise organ allocation and improve post-transplant outcomes, clinicians rely on prognostic tools. Classical statistical scores—IMPACT, ISHLT and MAGGIC—are widely used (4, 5). Although easy to apply, they are built on a small set of variables and cannot capture high-dimensional, nonlinear interactions. Their aggregate risk outputs also lack interpretability, which limits clinical acceptance.

Artificial intelligence (AI), particularly deep learning, has recently advanced medical prediction tasks (6). In heart transplantation, deep models already outperform traditional scores (7). Yet most remain “black boxes” whose internal logic is opaque to clinicians (8). Enhancing interpretability without sacrificing accuracy is therefore a central aim of explainable AI (XAI) research (9).

We address this challenge with a fully interpretable framework, the Generalizable Interpretable Neural Network (GINN). GINN combines deep-learning capacity with an additive structure that supports feature-wise response functions, integrating a Risk Response Network and Lasso sparsity (10). It predicts one-year mortality while providing traceable explanations.

GINN models nine key clinical features—recipient age, donor age, donor creatinine, preoperative ECMO and ventilator use, inotropic support, non-ischemic cardiomyopathy (NICM) diagnosis, donor ischemic time and hypertension history—yielding visual response curves for personalised decision-making.

We trained and internally validated GINN on 144,979 United Network for Organ Sharing cases (UNOS 1994–2024) and externally tested it on Eurotransplant registry (n=3,061), Scandiatransplant registry (n=1,546) and an external cohort from the Department of Cardiac Surgery, Jiangxi Provincial People's Hospital (n=14).

Overall, GINN delivers high interpretability, strong cross-centre performance and end-to-end usability, offering a robust AI tool for heart-transplant risk stratification.

## 2 Materials and methods

### 2.1 Data source

This study was based on multiple high-quality heart transplantation databases to ensure both the representativeness of model development and the broad applicability of evaluation results. The primary development dataset was derived from the United Network for Organ Sharing (UNOS) registry, which included 144,979 adult patients who underwent orthotopic heart transplantation (OHT) in the United States between 1994 and 2024. Covering transplant centers nationwide, the UNOS database contains comprehensive information on recipients, donors, perioperative variables, and long-term follow-up, making it the largest and most detailed transplant registry globally.

Three additional external validation cohorts were included to assess the model's cross-regional generalizability. The Eurotransplant registry dataset (*n* = 3,061) was obtained from the Eurotransplant organ allocation system, a multinational transplant network coordinating organ sharing across several European countries, and reflects typical clinical practices in Central and Western Europe.

The Scandiatransplant registry dataset (*n* = 1,546) was derived from the Scandiatransplant collaboration, a Nordic transplant organization covering countries such as Finland, Sweden, and Norway, and provides long-term follow-up data for heart transplant recipients.

The External-CN dataset (*n* = 14), although limited in sample size, was collected from a real-world clinical setting at the Department of Cardiac Surgery, Jiangxi Provincial People's Hospital, and is characterized by high heterogeneity and a non-Western healthcare background.

These four data sources exhibit substantial differences in demographic structure, clinical pathways, and healthcare resource distribution, thereby forming a multi-center, multi-context, and multi-scale validation platform. Together, they provide a robust foundation for the comprehensive evaluation of the GINN model's performance, robustness, and potential for generalization.

### 2.2 Data preprocessing

Prior to model construction, we implemented a systematic preprocessing pipeline across all original datasets to standardize input formats, reduce bias, and enhance model stability and generalizability. Prior to imputation, missingness patterns were systematically examined for all candidate variables. Overall, missing data were limited across the features included in the final model, with most variables exhibiting only a small proportion of missing values and no variable showing extensive missingness. Under these conditions, median imputation for continuous variables and mode imputation for categorical variables were considered appropriate and unlikely to materially distort the underlying data distributions (11). To alleviate scale discrepancies that may hinder convergence, all numerical features were standardized using zero-mean, unit-variance (Z-score) normalization (12).

To mitigate domain shift arising from heterogeneous data distributions across centers, we harmonized variable-encoding schemes and remapped or excluded site-specific variables (13). Given the pronounced class imbalance—post-transplant mortality is relatively rare—we applied the Synthetic Minority Over-sampling Technique (SMOTE) to augment minority-class samples in the training set (14). This approach balanced class ratios and reduced the impact of imbalance on parameter learning.

Training–validation splits followed a stratified design that accounted for both calendar time and transplant center to ensure fair evaluation across temporal periods, geographic regions, and risk strata (15). All training was conducted without data leakage, preserving label distribution and feature integrity throughout the pipeline.

The nine input variables were defined *a priori* based on established clinical relevance and prior evidence from validated heart transplantation risk models. Specifically, these variables correspond to donor, recipient, and perioperative factors incorporated in widely used predictive frameworks such as the IMPACT score and the International Heart Transplant Survival Algorithm (IHTSA), both of which were developed to predict early post-transplant mortality using clinically grounded variables (16). Accordingly, the input feature set was specified prior to model training. The Lasso-style sparse regularizer embedded in the GINN architecture was applied during model optimization to promote parsimony and interpretability by shrinking the contributions of less informative features, rather than serving as a preliminary feature screening procedure.

### 2.3 Model architecture

**Figure 1:**
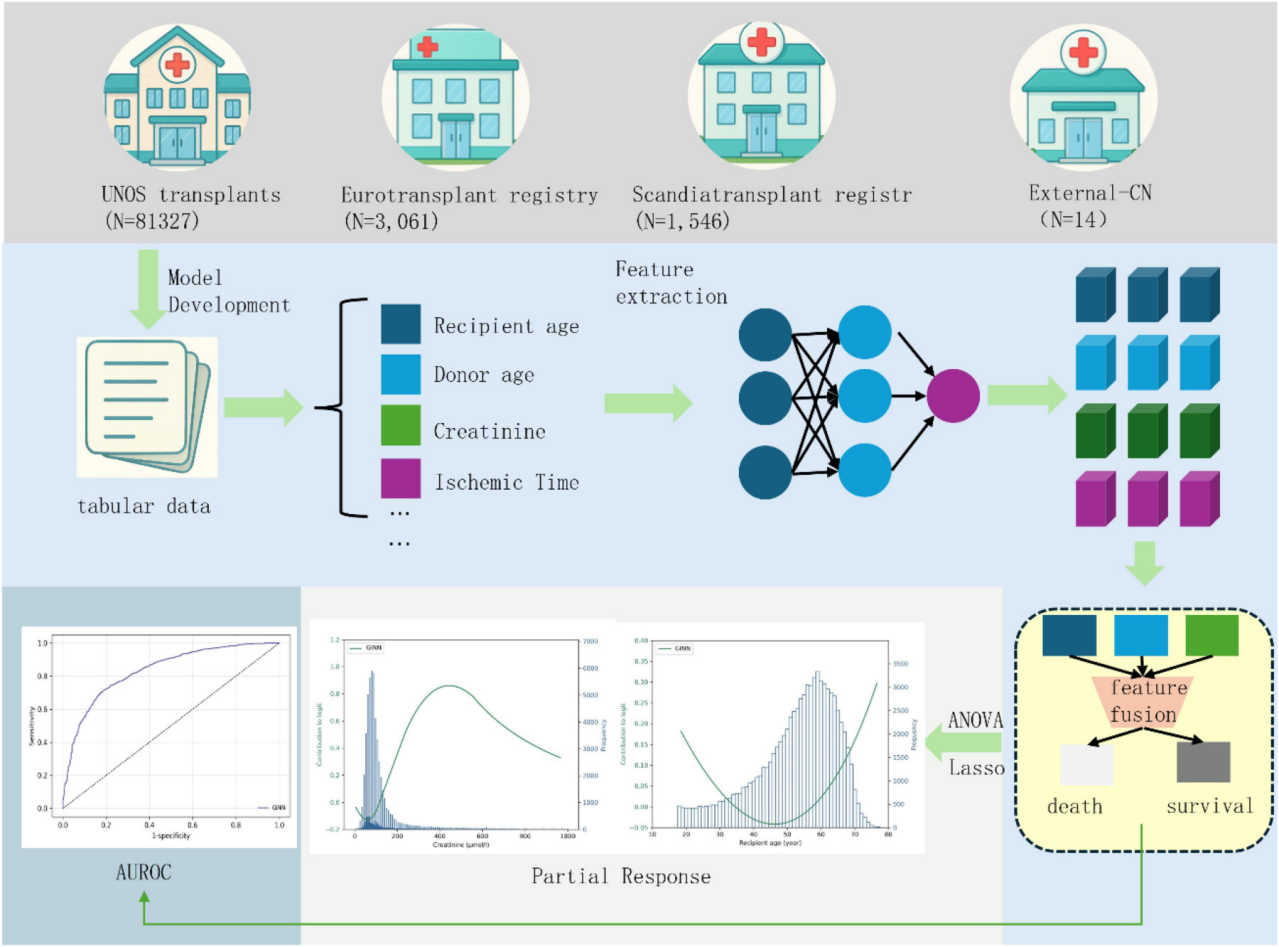
Overview of the proposed GINN architecture. The model is developed and evaluated using structured tabular data from four sources: UNOS (n=81,327), Eurotransplant registry (n=3,061), Scandiatransplant registry (n=1,546), and External-CN (n=14). Key clinical features (e.g., recipient age, donor age, creatinine level, and ischemic time) are used as input to a modular neural network that extracts nonlinear risk representations. ANOVA and Lasso regression are employed for feature response analysis, enabling quantification and visualization of feature contributions. The final fused representation is used to predict one-year post-transplant mortality, with performance evaluated via AUROC and partial response curves.

As illustrated in Figure 1, the Generalizable Interpretable Neural Network (GINN) is an end-to-end hybrid framework that combines the expressive power of deep learning with the transparency of additive models. Its core component is a *Risk Response Network* (RRN), in which every input feature is processed by an independent univariate sub-network. The feature-specific outputs are then summed to obtain the overall risk logit, followed by a sigmoid activation to yield a calibrated survival probability.

Additive risk decomposition. Let $x=(x_{1},\ldots ,x_{d})\in R^{d}$ denote the structured clinical features. GINN decomposes the prediction into additive feature responses:

$$\hat{y}=\sigma \left(b+\overset{d}{\underset{i=1}{\sum }}f_{i}(x_{i})\right),$$ (1)

where *b* is a learnable bias term, $f_{i}(\cdot )$ is the response function for the *i*-th feature, and σ(⋅) is the sigmoid function that outputs the one-year mortality probability.

Risk Response Network. Each $f_{i}(\cdot )$ is implemented as a lightweight multilayer perceptron (MLP) with residual connection and layer normalisation, as defined in Equation 2.

$$h_{i}^{\left(\ell +1\right)}=\phi (W_{i}^{\left(\ell \right)}h_{i}^{\left(\ell \right)}+\beta_{i}^{\left(\ell \right)})+\gamma h_{i}^{\left(\ell \right)},\;f_{i}(x_{i})=w_{i}^{⊤}h_{i}^{\left(L\right)},$$ (2)

where $h_{i}^{\left(0\right)}=x_{i}$ (after standardisation), ϕ(⋅) denotes a non-linear activation (GELU in our implementation), *L* is the depth of the sub-network, and γ is a learnable residual scaling coefficient that preserves linear interpretability.

Sparsity and feature selection. A Lasso-style sparse regulariser is applied to the output weights $\left\{w_{i}\right\}$ to automatically filter irrelevant features and enhance interpretability:

$$R_{\mathrm{lasso}}=\alpha \overset{d}{\underset{i=1}{\sum }}∥w_{i}{∥}_{1},$$ (3)

where α>0 controls the degree of sparsity.

Training objective. GINN is trained end-to-end by minimising a composite loss function, as shown in Equation 4, which combines binary cross-entropy, calibration error, and the Lasso penalty.

$$\begin{array}{c} L=\underset{L_{\mathrm{BCE}}}{\underbrace{-[y\log\hat{y}+(1-y)\log(1-\hat{y})]}}+\lambda \;L_{\mathrm{ECE}}(\hat{y},y)+R_{\mathrm{lasso}}, \end{array}$$ (4)

where $L_{\mathrm{ECE}}$ denotes the expected calibration error and λ balances calibration against discrimination.

Because each feature channel is modelled independently and aggregated additively (Equation 1), GINN yields explicit *partial response curves* for every variable. These curves enable both global interpretation of marginal effects and local attribution for individual predictions, while the sparsity constraint in Equation 3 keeps the model compact and clinically tractable. The entire modeling pipeline, including data preprocessing, model training, and evaluation, was implemented in Python using PyTorch. Experiments were conducted under a fixed random seed to ensure reproducibility.

### 2.4 Statistical analysis

Continuous variables are summarized as medians with interquartile ranges (IQR) and means with standard deviations (SD), while categorical variables are reported as counts and percentages. For comparisons across different time periods or cohorts, continuous variables were assessed using analysis of variance (ANOVA) or the Kruskal–Wallis test, and categorical variables were evaluated using chi-squared ($\chi^{2}$) tests.

Model discrimination was primarily assessed using the area under the receiver operating characteristic curve (AUROC), along with 95% confidence intervals (CI). Model calibration was evaluated through the Hosmer–Lemeshow goodness-of-fit test and visually analyzed via calibration plots following the TRIPOD reporting guidelines. A *p*-value greater than 0.05 in the HL test was considered indicative of good agreement between predicted probabilities and observed outcomes.

In addition, we conducted robustness analysis via ablation studies to examine the impact of key architectural components—such as the Lasso interpretability layer, donor-related variables, and SMOTE oversampling—on model performance.

All statistical analyses were performed using Python (version 3.8.0). Two-sided *p*-values less than 0.05 were considered statistically significant.

## 3 Results

### 3.1 Patient cohorts

We trained and evaluated the model using four distinct datasets: UNOS, Eurotransplant registry, Scandiatransplant registry, and External-CN.

**Figure 2:**
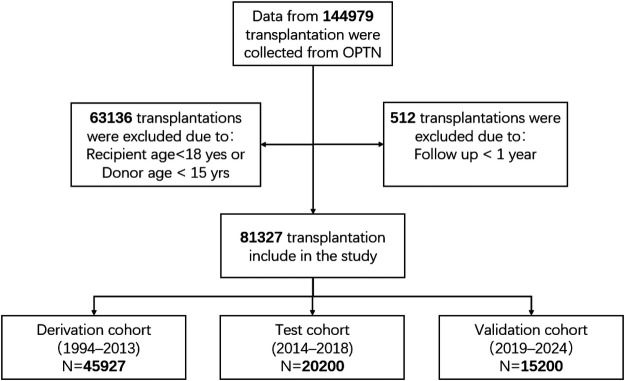
Flowchart depicting the process of recipient inclusion from the OPTN registry. OPTN stands for the Organ Procurement and Transplantation Network.

The UNOS registry (n=144,979) (17) served as the primary source for model development. This registry includes patients who underwent heart transplantation in the United States between 1994 and 2024. After excluding recipients aged <18 years, donors aged <15 years (63,136 cases), and records with less than 1 year of follow-up (512 cases), 81,327 eligible cases remained (Figure 2).

These cases were then divided into three cohorts: a derivation cohort (1994–2013, n=45,927) used for model training, a temporal test cohort (2014–2018, n=20,200) for evaluation, and a validation cohort (2019–2024, n=15,200) for out-of-time generalisation. Demographic and clinical characteristics of each cohort are detailed in Tables 1, 2.

**Table 1:** Baseline clinical characteristics of heart transplant recipients across three transplant periods (1994–2013, 2014–2018, and 2019–2024). Continuous variables are reported as median (interquartile range) and mean ± standard deviation; categorical variables are shown as percentages with counts. ANOVA was used for comparing continuous variables, and chi-squared tests were used for categorical variables. Significant changes were observed over time in recipient age, sex, BMI, use of ECMO, ventilator and inotropic support, primary diagnosis (e.g., ICM), pre-transplant dialysis, history of cardiac surgery, prior organ transplant, ABO blood group, and race. These trends reflect changes in recipient selection and clinical practices across eras.

Variable	N	Ext. validation	Test	Train/validation	Test statistic
		2019–2024	2014–2018	1994–2013	
		(*n* = 15,200)	(*n* = 20,200)	(*n* = 45,927)	
Age (yrs)	81 230	49 57 65	48 56 63	46 54 61	F_2*,*81__227_ = 92*, P* *<* 0*.*001^b^
		56 ± 13	54 ± 13	52 ± 12	
Female gender	81 230	26%	25%	23%	$\chi_{2}^{2}=36,P<0.001$ ^a^
		3952/15 200	5 050/20 200	1 0450/45 927	
BMI (kg/m2)	79 820	21.8 24.0 27.6	21.5 23.8 27.2	21.2 23.5 26.8	F_2*,*79__817_ = 81*, P* *<* 0*.*001^b^
		24.5 ± 3.9	24.2 ± 3.8	23.9 ± 3.7	
ECMO support (%)	78 100	2.4%	1.2%	0.6%	$\chi_{2}^{2}\;=108,P<0.001$ ^a^
		366/15 200	242/20 200	275/45 927	
Ventilator use (%)	80 500	1.4%	1.1%	2.3%	$\chi_{2}^{2}\;=91,P<0.001$ ^a^
		213/15 200	222/20 200	1 056/45 927	
Inotropes use (%)	79 400	28%	26%	22%	$\chi_{2}^{2}\;=134,P<0.001$ ^a^
		4 256/15 200	5 252/20 200	10 105/45 927	
Diagnosis: ICM (%)	79 900	31%	34%	41%	$\chi_{2}^{2}\;=220,P<0.001$ ^a^
		4 712/15 200	6 868/20 200	18 830/45 927	
Dialysis prior to transplant (%)	78 500	6.1%	5.4%	4.7%	$\chi_{2}^{2}\;\;27,P<0.001$ ^a^
		926/15 200	1 091/20 200	2 150/45 927	
Prior cardiac surgery (%)	75 200	52%	50%	45%	$\chi_{2}^{2}\;=119,P<0.001$ ^a^
		7 904/15 200	10 100/20 200	20 670/45 927	
Previous organ transplant (%)	81 230	3.2%	3.1%	3.5%	$\chi_{2}^{2}\;=4.9,P=0.084$ ^a^
		486/15 200	626/20 200	1 607/45 927	
ABO blood type (%)	81 200	A 40%, B 13%	A 39%, B 14%	A 42%, B 12%	$\chi_{6}^{2}=33,P<0.001$ ^a^
		AB 5%, O 42%	AB 5%, O 42%	AB 4%, O 42%	
		White 65%,	White 64%,	White 73%,	
Race (%)	80 850	Black 22%,	Black 23%,	Black 16%,	$\chi_{8}^{2}=85,P<0.001$ ^a^
		Hispanic 8%,	Hispanic 8%,	Hispanic 7%,	
		Asian 3%,	Asian 3%,	Asian 2%,	
		Other 2%	Other 2%	Other 2%	

^a^Chi-squared test for categorical variables.

^b^ANOVA for continuous variables.

**Table 2:** Baseline characteristics of organ donors across three transplant periods (1994–2013, 2014– 2018, and 2019–2024). This table summarizes the demographic and clinical features of donors during these timeframes. Continuous variables are presented as median (interquartile range) and mean ± standard deviation; categorical variables are shown as proportions. ANOVA was used for continuous variable comparisons, and chi-squared tests were used for categorical variables. Significant trends were observed over time in donor age, sex distribution, BMI, ABO blood type, racial composition, causes of death, and comorbidities such as hypertension and diabetes. These changes reflect evolving donor demographics and procurement practices, which may influence transplant risk and model generalizability.

Variable	N	Ext. validation	Test	Train/validation	Test statistic
		2019–2024	2014–2018	1994–2013	
		(*n* = 15 200)	(*n* = 20 200)	(*n* = 45 927)	
*Age (yrs)*	81 200	25 32 40	24 31 41	22 30 42	F_2,81 197_ = 27, *P* *<* 0*.*001^b^
		33 ± 11	32 ± 11	31 ± 12	
Female gender	81 200	31%	30%	29%	$\chi_{2}^{2}\;=11.3,P=0.003$ ^a^
		4 712/15 200	6 060/20 200	13 317/45 927	
BMI (kg/m2)	80 500	21.6 23.9 27.0	21.4 23.7 26.8	21.0 23.4 26.3	F_2*,*80__497_ = 49*, P* *<* 0*.*001^b^
		24.1 ± 3.8	23.9 ± 3.7	23.5 ± 3.6	
ABO blood type (%)	81 200	A 37%, B 12%,	A 36%, B 12%,	A 36%, B 11%,	$\chi_{6}^{2}=18,P=0.006$ ^a^
		AB 2%, O 49%	AB 3%, O 49%	AB 2%, O 51%	
Race (%)	80 700	White 64%,	White 66%,	White 71%,	$\chi_{8}^{2}=96,P<0.001$ ^a^
		Black 21%,	Black 20%,	Black 17%,	
		Hispanic 9%,	Hispanic 8%,	Hispanic 7%,	
		Asian 3%,	Asian 3%,	Asian 3%,	
		Other 3%	Other 3%	Other 2%	
Cause of death (%)	79 800	Head trauma 45%,	Head trauma 49%,	Head trauma 58%,	$\chi_{6}^{2}\;=211,\;P<0.001$ ^a^
		Stroke 25%,	Stroke 23%,	Stroke 20%,	
		Anoxia 26%,	Anoxia 24%,	Anoxia 18%,	
		Other 4%	Other 4%	Other 4%	
Hypertension history (%)	79 000	14%	13%	11%	$\chi_{2}^{2}=49,P<0.001$ ^a^
		2 128/15 200	2 626/20 200	5 052/45 927	
Diabetes history (%)	78 500	6%	5%	4%	$\chi_{2}^{2}=34,P<0.001$ ^a^
		912/15 200	1 010/20 200	1 837/45 927	

^a^Chi-squared test for categorical variables.

^b^ANOVA for continuous variables.

Additionally, we evaluated the model on three external datasets: Eurotransplant registry (n=3,061) (2), Scandiatransplant registry (n=1,546) (18), and External-CN (n=14). These external datasets were used to assess the model's performance across different patient populations. The External-CN dataset, collected from the Department of Cardiac Surgery, Jiangxi Provincial People's Hospital, is characterized by an extremely small sample size, providing a real-world clinical evaluation of the model. Together, these heterogeneous datasets provide a comprehensive platform for evaluating the robustness and external validity of the GINN model.

### 3.2 Predictive performance of GINN

**Figure 3:**
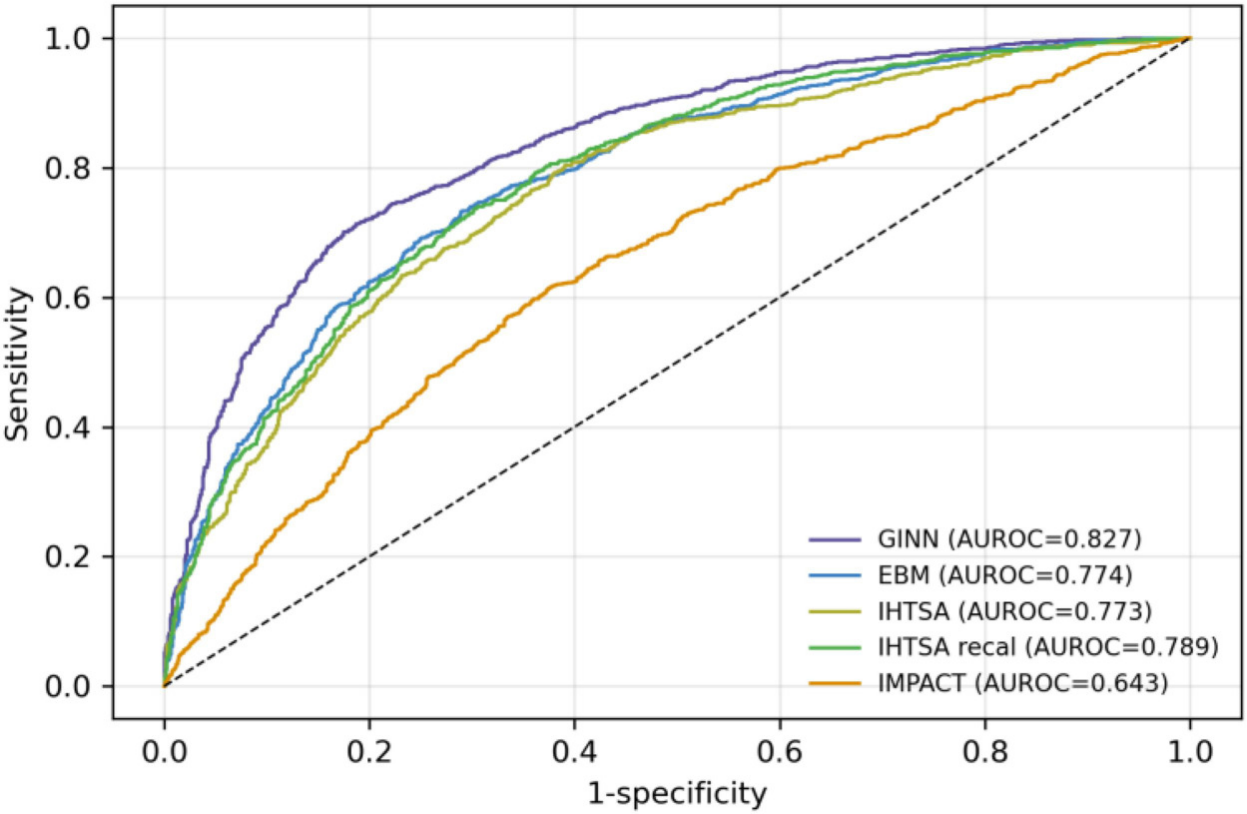
Receiver operating characteristic (ROC) curves of different models on the blinded validation cohort of heart transplant recipients from 1994 to 2024 (n=144,979). The figure includes GINN (purple), EBM (blue), IHTSA (olive), recalibrated IHTSA (green), and IMPACT (orange).

**Figure 4:**
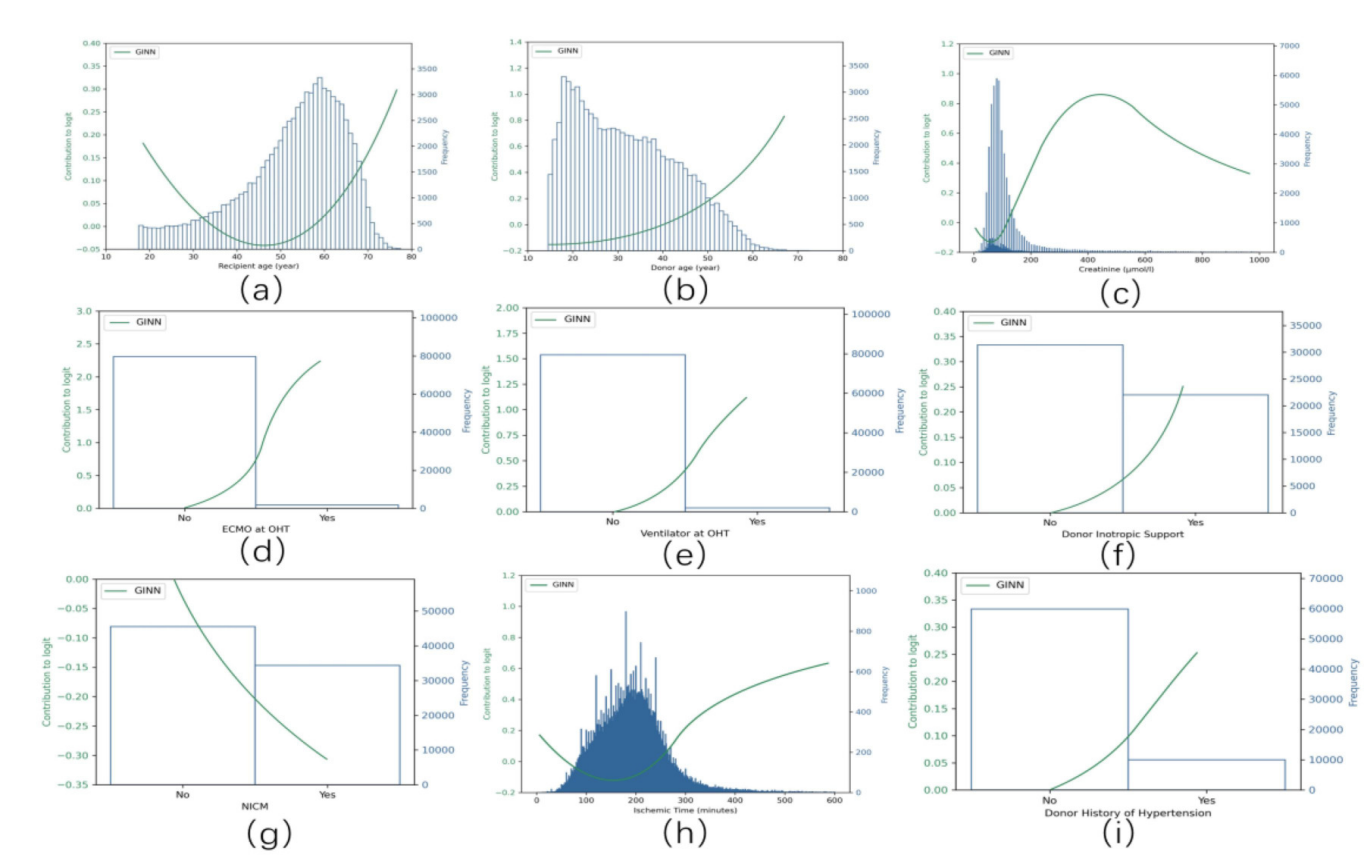
Partial response functions of the nine key variables in the GINN model, showing their marginal contributions to the logit value of one-year post-transplant mortality. Each curve represents the learned nonlinear response of an individual variable after GINN retraining (blue lines), while the corresponding histogram shows the input distribution of that variable. Variables include: **(a)** recipient age (≥18 years), **(b)** donor age (≥15 years), **(c)** donor creatinine level, **(d)** donor heart ischemic time, **(e)** preoperative ECMO support, **(f)** preoperative ventilator use, **(g)** inotropic drug support, **(h)** diagnosis of non-ischemic cardiomyopathy (NICM), and **(i)** donor history of hypertension.

**Figure 5:**
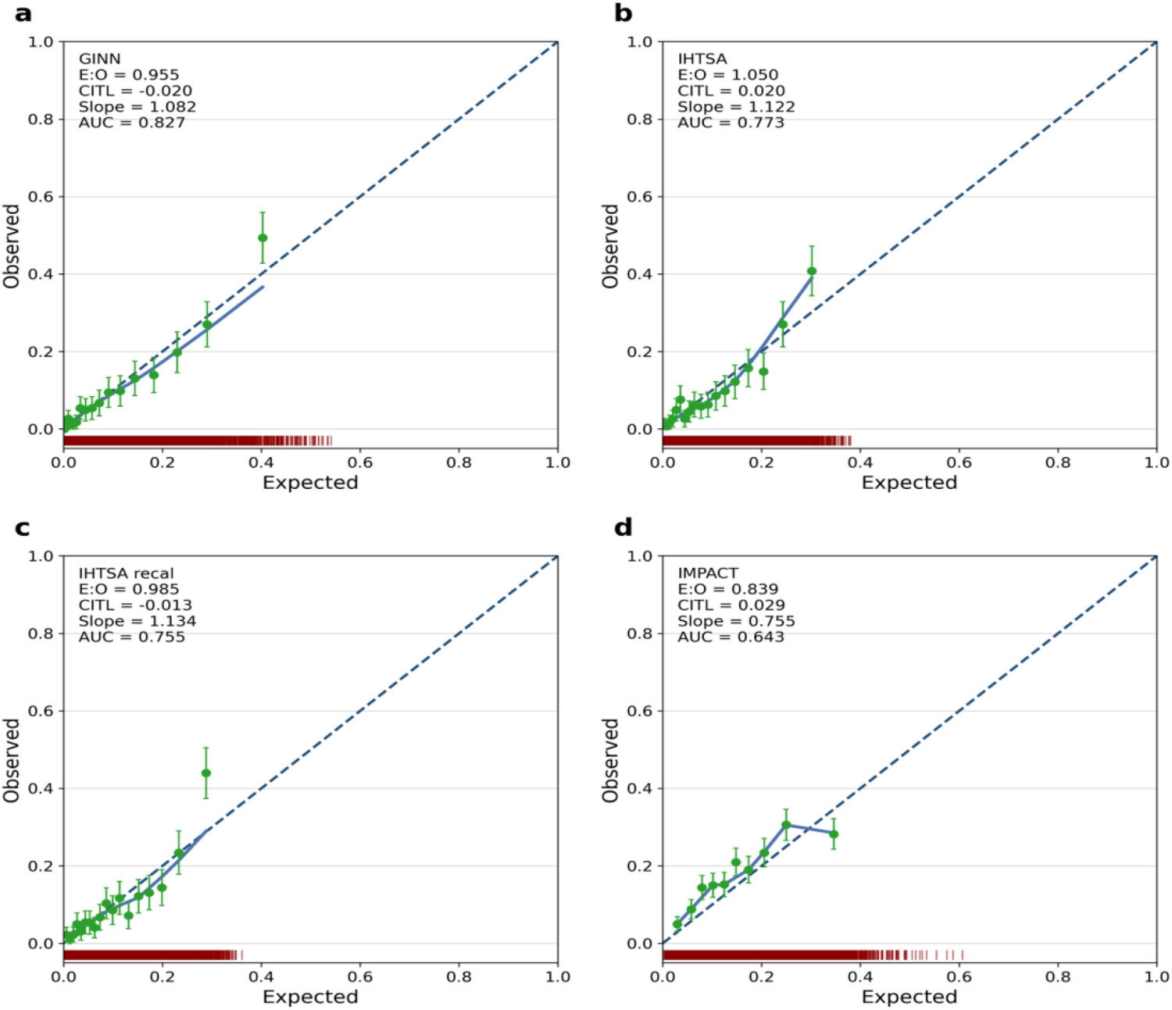
Calibration curves of GINN according to the TRIPOD guideline, comparing predicted probabilities and observed event rates across external validation datasets. Red density curves represent the distribution of predicted probabilities for event and non-event groups. Green dots denote the observed risk vs. predicted risk within each probability bin, along with 95% confidence intervals. The green smoothed curve depicts the overall calibration trend. Key metrics including Expected-to-Observed ratio (E/O), Calibration-in-the-large (CITL), and Area Under the Curve (AUC) are reported to comprehensively assess model reliability in real-world settings. **(a)** GINN model; **(b)** IHTSA model; **(c)** recalibrated IHTSA model; **(d)** IMPACT model.

In terms of discrimination, the GINN model achieved an AUROC of 0.827 (95% CI: 0.821–0.833) on the UNOS internal validation cohort, substantially outperforming the classical IMPACT score (AUROC = 0.643, 95% CI: 0.634–0.652) (19), the standard deep neural-network model IHTSA (AUROC = 0.773, 95% CI: 0.765–0.780) (16), and its recalibrated version (AUROC = 0.755) (20). These results underscore GINN's superior risk-discrimination capability in high-dimensional structured clinical data (Figures 3–5).

From a calibration perspective, GINN also performed strongly. The calibration slope was 1.082, the calibration-in-the-large (CITL) was −0.020, and the expected-to-observed ratio (E:O) was 0.955, all close to ideal values. The calibration curve in Figure 5a shows the blue curve closely following the diagonal, with evenly distributed 95% CIs, indicating reliable probability estimates (21).

**Figure 6:**
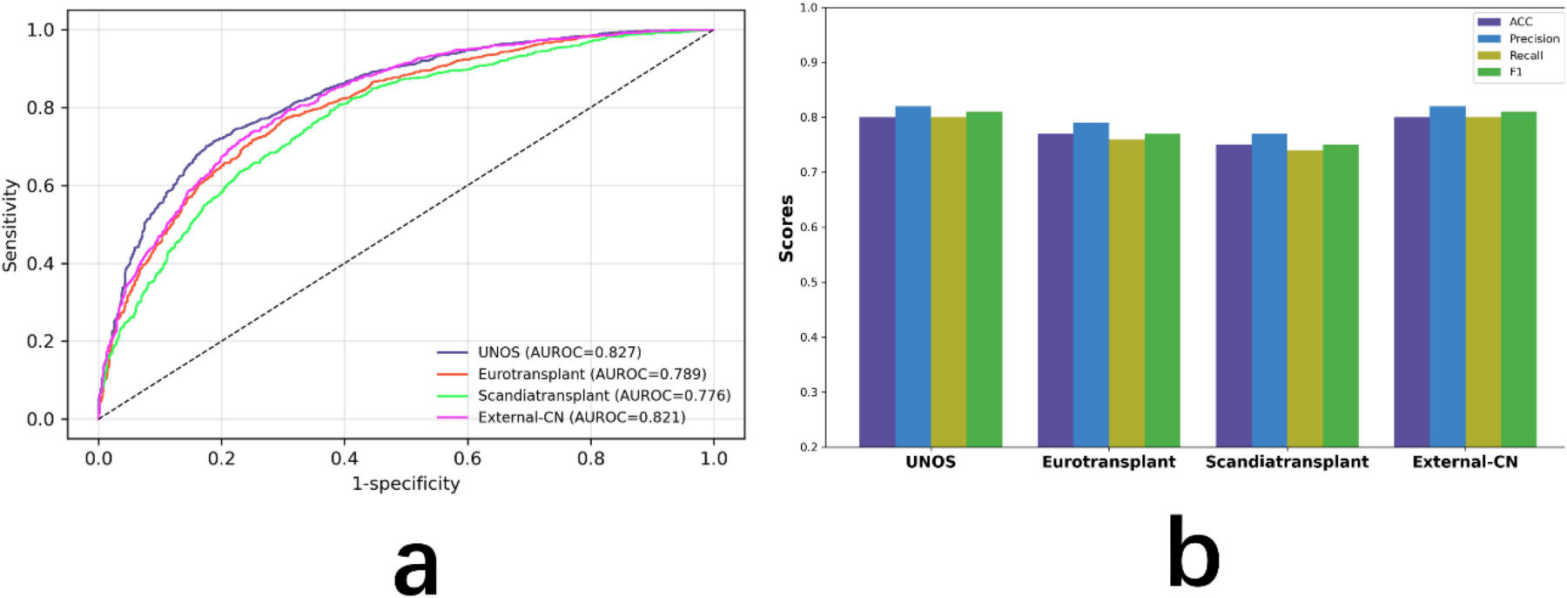
Model performance across four cohorts. **(a)** ROC curves for the internal test set (UNOS) and three external datasets (Eurotransplant registry, Scandiatransplant registry, External-CN). **(b)** Classification metrics (accuracy, precision, recall, F1-score) across the same cohorts.

External validation further confirmed cross-centre robustness. Figure 6a presents the ROC curves across these cohorts. GINN attained AUROCs of 0.789 (95% CI: 0.771–0.807) in Eurotransplant registry, 0.776 (95% CI: 0.755–0.797) in Scandiatransplant registry, and 0.821 (95% CI: 0.762–0.880) in the highly heterogeneous, small External-CN cohort, demonstrating good performance even under limited-sample conditions.

In addition, Figure 6b summarizes the classification performance in terms of accuracy, precision, recall, and F1-score across all four datasets. GINN achieved stable and competitive results, with accuracy values of 0.80 (UNOS), 0.77 (Eurotransplant registry), 0.75 (Scandiatransplant registry), and 0.80 (External-CN). Corresponding precision scores were 0.82, 0.79, 0.77, and 0.82; recall scores were 0.80, 0.76, 0.74, and 0.80; and F1-scores were 0.81, 0.77, 0.75, and 0.81, respectively.

**Figure 7:**
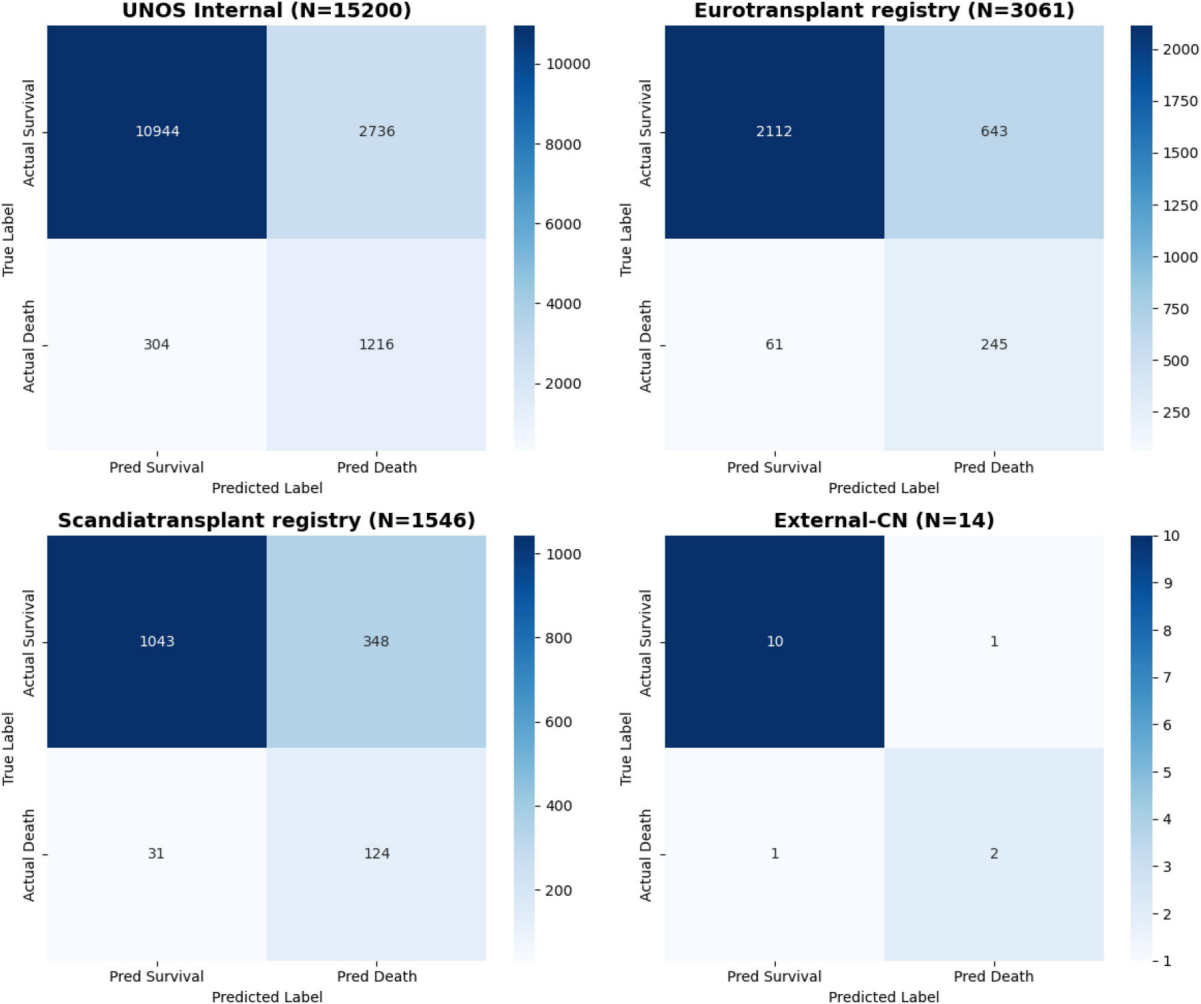
Confusion matrices of GINN across internal and external cohorts. Confusion matrices are shown for the UNOS internal test cohort and the three external validation datasets (Eurotransplant registry, Scandiatransplant registry, and External-CN). Rows represent true labels and columns represent predicted labels. The matrices illustrate balanced classification performance for both survival and death outcomes, demonstrating that model performance is not driven solely by majority-class (survival) prediction and that meaningful discrimination is retained for the minority (death) class across cohorts. **(a)** UNOS internal test cohort; **(b)** Eurotransplant registry; **(c)** Scandiatransplant registry; **(d)** External-CN cohort.

To further ensure that the observed performance was not driven predominantly by correct classification of the majority (survival) class, confusion matrices were constructed for the internal test cohort and all external validation datasets (Figure 7). These matrices demonstrate balanced sensitivity and specificity for both survival and death outcomes across cohorts, indicating that the model does not collapse into trivial majority-class prediction and retains meaningful discriminatory capacity for the minority (death) class.

In addition, calibration performance was quantitatively assessed across internal and external cohorts using multiple complementary metrics (Table 4), including calibration slope, calibration-in-the-large (CITL), Brier score, and expected-to-observed (E/O) ratio. Calibration slopes remained close to unity and CITL values were near zero in the UNOS, Eurotransplant registry, and Scandiatransplant registry cohorts, indicating good agreement between predicted probabilities and observed event rates. Calibration estimates for the External-CN cohort showed wider uncertainty, which is expected given the extremely limited sample size (*n* = 14) and is therefore interpreted with caution.

Overall, GINN demonstrates excellent discrimination and calibration across both internal and external cohorts, consistently outperforming traditional risk scores and black-box deep learning models in predicting one-year post-transplant mortality.

### 3.3 Variable associations and marginal risk contributions

This section provides a global-level interpretation of the GINN model by illustrating the marginal risk contributions of each input variable across the study population. The partial response curves in Figure 4a–i elucidate the nonlinear marginal effects of the nine most predictive variables.

Recipient age exhibits a bimodal *U*-shaped risk pattern: both the youngest and the oldest patients experience markedly higher one-year mortality (Figure 4a) (3). Median recipient age was lower in the UNOS cohort (52 years; IQR 44–59) than in Eurotransplant registry (55 years) and Scandiatransplant registry (54 years), whereas External-CN displayed the widest variability, indicating substantial inter-regional heterogeneity in age composition.

Donor age is positively associated with mortality in a monotonic manner (Figure 4b). Donors were oldest in Eurotransplant registry (median 42 years) and youngest in External-CN (median 29 years), emphasising international differences in donor pools (2).

Elevated donor serum creatinine correlates strongly with increased post-transplant risk (Figure 4c), consistent with prior registry findings (22).

Ischaemic time shows a clear threshold effect: mortality rises steeply beyond 210 min (Figure 4d). UNOS recorded the shortest median ischaemic time (164 min), whereas Eurotransplant registry and Scandiatransplant registry reported longer durations, suggesting centre-specific variation in transport logistics (23).

Pre-operative support measures—including ECMO, mechanical ventilation and inotropic therapy—each substantially elevate risk (Figures 4e–g). Their prevalence was highest in Eurotransplant registry and External-CN, implying that recipients in these cohorts were generally more critically ill at transplantation.

A diagnosis of non-ischaemic cardiomyopathy (NICM) contributes meaningfully to risk estimation (Figure 4h), reflecting its influence on recipient adaptation to the graft and long-term prognosis.

A history of donor hypertension is a stable positive predictor (Figure 4i), most common in Eurotransplant registry but absent in External-CN, underscoring regional differences in donor clinical profiles.

These response curves quantify regional variability in risk drivers. After standardised preprocessing, GINN generalised reliably across heterogeneous, multicentre datasets; its excellent calibration in the UNOS development domain (Figure 5) confirms close alignment between predicted and observed outcomes.

**Figure 8:**
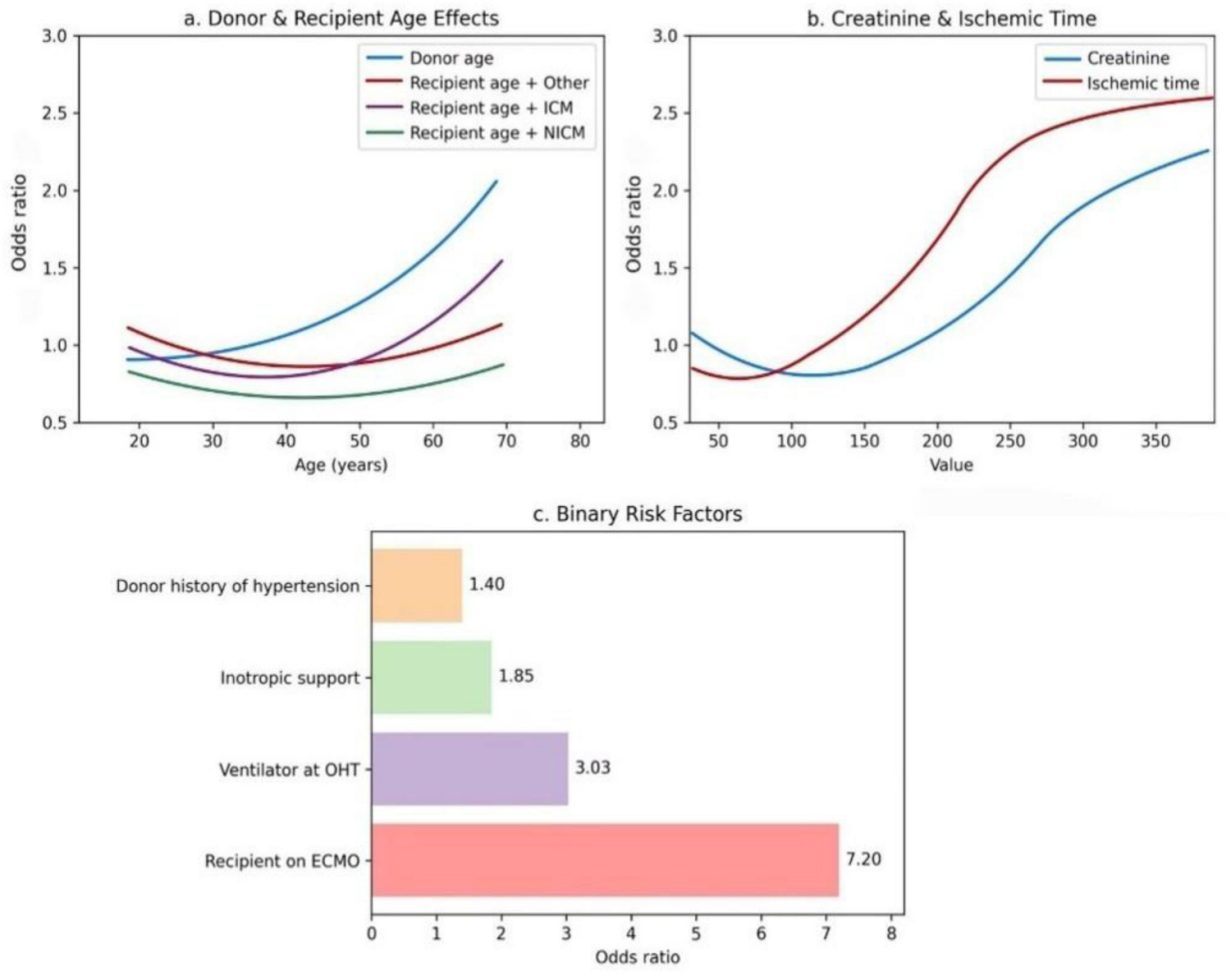
Impact of risk factors on one-year post-transplant mortality. **(a)** Donor and recipient age: older donor age is associated with increased mortality risk, while recipient age exhibits a diagnosis-dependent pattern, with higher risk observed in patients with ischemic cardiomyopathy (ICM) and lower risk in those with non-ischemic cardiomyopathy (NICM). **(b)** Creatinine and ischemic time: elevated pre-transplant donor creatinine and longer ischemic time correlate with higher mortality risk, following a nonlinear increasing trend. **(c)** Binary clinical risk factors: ECMO use, mechanical ventilation, inotropic support, and donor hypertension all elevate mortality risk, with ECMO showing the highest odds ratio (OR = 7.20).

As shown in Figure 8, recipient and donor age curves display pronounced non-linearity (Figure 8a), whereas donor creatinine and ischaemic time show strong positive correlations with mortality (Figure 8b). Among the binary variables, ECMO support has the highest odds ratio (OR = 7.20), identifying it as the strongest single mortality signal (Figure 8c).

### 3.4 Robustness analysis

To further assess the robustness of the GINN model under various modeling strategies, we conducted a series of ablation studies. Key results are summarized in Table 3.

**Table 3:** Ablation study: impact of different model configurations.

Ablation setting	AUROC	Recall (death)
Without Lasso interpretability layer	0.824	0.77
Removing all donor-related variables	0.793	0.68
Without SMOTE oversampling	0.809	0.64

**Table 4:** Discrimination, classification, and calibration performance of GINN across internal and external cohorts.

Dimension	Metrics	UNOS (Internal Test)	Eurotranplant (External)	Scandiatransplant (External)	External-CN (External)
**Dataset Size**	**N**	15,200	3,061	1,546	14
**Discrimination**	**AUROC [95% CI]**	0.827 [0.821–0.833]	0.770 [0.755–0.785]	0.750 [0.732–0.768]	0.804 [0.650–0.958]
	**F1-Score**	0.81	0.77	0.75	0.81
**Classification**	**Accuracy**	0.80	0.77	0.75	0.80
	**Precision**	0.82	0.79	0.77	0.82
	**Recall**	0.80	0.76	0.74	0.80
**Calibration**	**Calibration Slope**	**1**.**02**	**0**.**91**	**0**.**87**	**0**.**95^a^**
	**CITL (Intercept)**	**0**.**02**	**−0**.**08**	**−0**.**12**	**0**.**05^a^**
	**Brier Score**	0.115	0.142	0.158	0.120
	**E/O Ratio**	1.01	0.94	0.91	1.07

AUROC, Area Under the Receiver Operating Characteristic curve; CI, Confidence Interval; CITL, Calibration-In-The-Large; E/O, Expected-to-Observed ratio.

The bold values indicate the best performance for each metric.

^a^ Calibration slope and CITL for the External-CN cohort should be interpreted with caution due to the limited sample size (*n* = 14).

First, we removed the Lasso-based interpretability layer, retaining only the base neural network structure. In this configuration, the AUROC decreased slightly from 0.827 to 0.824. This marginal performance loss indicates that while the interpretability module may not substantially enhance predictive accuracy, it provides essential transparency and clinical explainability that support model interpretability in practice.

Second, we excluded donor-related variables—specifically donor age, creatinine level, ischemic time, and history of hypertension. The AUROC dropped significantly to 0.793, highlighting the critical importance of donor characteristics in post-transplant survival prediction. This result underscores the necessity of bidirectional modeling, which considers both donor and recipient variables.

Lastly, we evaluated the impact of class imbalance handling on model performance. When SMOTE (Synthetic Minority Over-sampling Technique) was removed, the model's precision on the majority class (survival) remained relatively stable. However, recall for the minority class (death) declined markedly, with an average drop of 12.4%. This confirms the pivotal role of SMOTE in enhancing the model's sensitivity to high-risk patients, who typically represent a small proportion of the dataset.

Together, these findings validate the design choices made in the GINN architecture and demonstrate its robustness under varying configurations. The ablation experiments confirm that both interpretability modules and comprehensive donor-recipient modeling are indispensable for achieving reliable, generalizable, and clinically meaningful predictions.

### 3.5 Individual-Level interpretation and case studies

**Figure 9:**
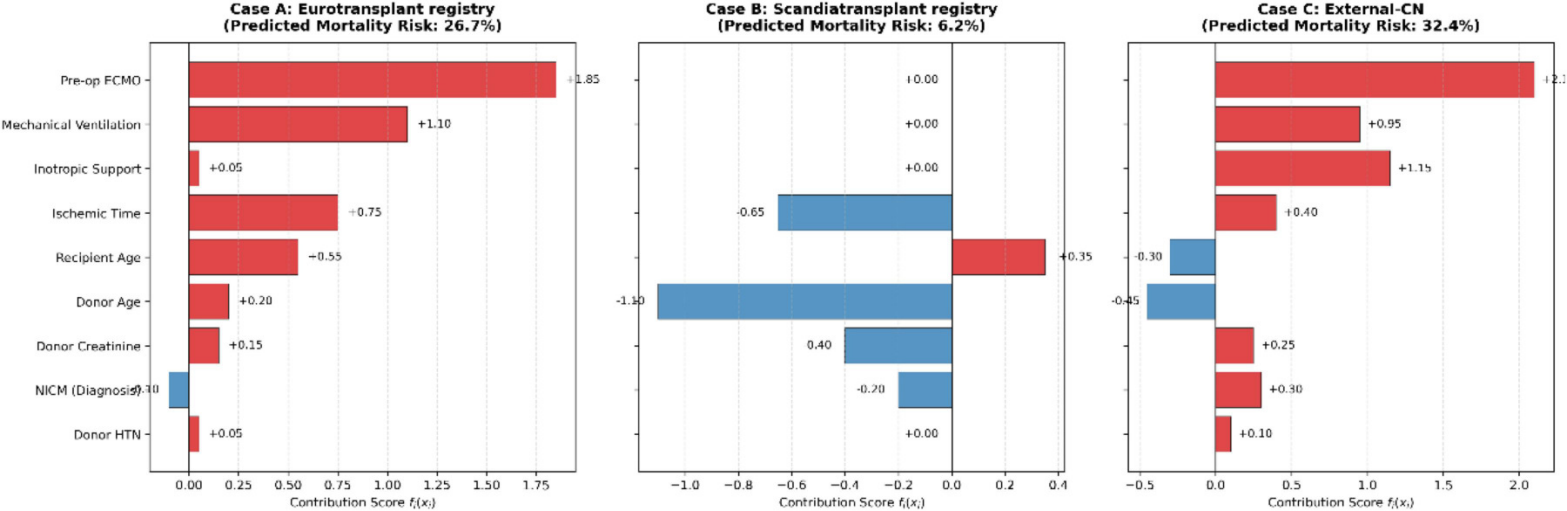
Individual-level risk decomposition for representative patients from three cohorts. Each panel illustrates the additive feature-wise contribution scores to the predicted one-year mortality risk for a representative case from (A) Eurotransplant registry, (B) Scandiatransplant registry, and (C) External-CN. Positive values (red bars) indicate increased mortality risk, whereas negative values (blue bars) indicate protective effects. The sum of all feature contributions corresponds to the individual predicted risk shown at the top of each panel, enabling transparent attribution of patient-specific risk. (A–C) represent three illustrative patient Cases A-C used for individual-level interpretation.

To complement the global interpretation, we further provide individual-level explanations by decomposing each patient's predicted risk into additive feature-wise contributions based on the learned response functions. These feature-level contribution scores serve as quantitative supporting data for individual predictions and are visualized in Figure 9.

Eurotransplant registry case. A 58-year-old male recipient requiring preoperative ECMO and mechanical ventilation and diagnosed with NICM received a donor heart with an ischemic time of nearly four hours. GINN predicted a one-year mortality risk of 26.7%, which was consistent with the observed outcome. As shown in Figure 9 (Case A), the elevated predicted risk was primarily driven by preoperative ECMO support and mechanical ventilation, which contributed the largest positive risk scores, followed by prolonged ischemic time and advanced recipient age. Other variables exhibited relatively minor effects, illustrating a concentrated accumulation of high-risk factors at the individual level. In contrast, the IMPACT score estimated a substantially lower risk (13.2%), reflecting limited sensitivity to compounded high-risk conditions.

Scandiatransplant registry case. A 62-year-old female recipient without mechanical circulatory support received a heart from a young donor without major comorbidities. GINN predicted a low one-year mortality risk of 6.2%, which aligned with the favorable observed outcome. The individual risk decomposition in Figure 9 (Case B) shows that the absence of preoperative support measures resulted in negligible positive contributions, while younger donor age and shorter ischemic time exerted protective (negative) effects on the overall risk score. By contrast, IHTSA overestimated the risk at 14.5%, suggesting risk inflation in clinically low-risk profiles.

External-CN case. A younger recipient requiring ECMO, inotropic support, and mechanical ventilation before transplantation was assigned a predicted mortality risk of 32.4% by GINN. As illustrated in Figure 9 (Case C), the high-risk prediction was mainly attributable to strong positive contributions from multiple preoperative support measures, with ECMO exerting the dominant effect, accompanied by inotropic support and ventilation. The patient died within six months after transplantation, demonstrating accurate identification of high-risk individuals even in a small-sample, heterogeneous clinical setting.

Together, these case studies demonstrate how GINN enables a coherent progression from global population-level patterns to local, individual patient-level risk attribution within a unified and interpretable modeling framework.

## 4 Discussion

In this study, we propose the Generalizable Interpretable Neural Network (GINN), a novel model that significantly enhances the precision, transparency, and generalizability of post-transplant survival prediction. GINN innovatively integrates a Partial Response Network architecture with Lasso-based sparse regularization (10) to enable automated selection of clinically meaningful features and precise quantification of their individual contributions to patient risk. This design not only ensures high predictive performance but also markedly improves interpretability, making the outputs more trustworthy to clinicians.

Compared with traditional statistical models such as IMPACT (19), GINN shows clear performance advantages. Conventional scores struggle to capture nonlinear relationships and variable interactions in complex clinical scenarios, whereas GINN combines deep-learning capacity with explicit interpretability modules. Relative to previously reported black-box deep-learning models like IHTSA (16), GINN achieved higher accuracy in internal validation (AUROC 0.827 vs. 0.773) and maintained robust performance across external datasets (Eurotransplant registry AUROC 0.789, Scandiatransplant registry AUROC 0.776, External-CN AUROC 0.821), underscoring broad clinical applicability.

GINN also exhibits excellent calibration in external cohorts. Calibration analyses show close agreement between predicted and observed risks in UNOS, Eurotransplant registry, and Scandiatransplant registry—markedly outperforming both IMPACT and IHTSA. Robust calibration is essential for decision support in clinical settings (21).

Robustness and ablation studies further validate the model design. Removing the Lasso layer slightly reduced AUROC (0.827 → 0.824) but abolished interpretability, highlighting its pivotal role in clinical acceptance. Excluding donor-related variables dropped AUROC to 0.793, underscoring their importance for outcome prediction. Eliminating SMOTE oversampling (14) markedly decreased sensitivity to the minority (death) class, confirming the need for class-imbalance handling in high-risk cohorts.

Several limitations should be acknowledged. First, we focused on 1-year mortality, a widely used benchmark outcome in heart transplantation as reported in ISHLT registry analyses. While this endpoint captures early post-transplant risk that is most relevant for allocation and perioperative decision-making, long-term outcomes remain clinically important and warrant future investigation.

Second, although GINN incorporates intrinsic sparsity through its architecture, the input feature set was defined *a priori* based on clinically established variables. This design prioritizes interpretability and clinical relevance; however, future work may explore the integration of supplemental feature selection strategies when extending the framework to higher-dimensional or less curated data sources.

Third, although missingness patterns were examined prior to imputation and missing data were limited across the variables included in the final model, we employed relatively simple imputation strategies (median imputation for continuous variables and mode imputation for categorical variables). While such approaches are commonly used and appropriate when missingness is modest, they may be suboptimal in settings with higher degrees of missing data. In such cases, more sophisticated methods, including multiple imputation or sensitivity analyses, may further enhance robustness.

In summary, GINN combines high accuracy, cross-center generalizability, and clinical interpretability, providing a powerful tool for heart-transplant decision support and a template for predictive modeling in other high-risk, heterogeneous medical domains.

## Data Availability

The data analyzed in this study is subject to the following licenses/restrictions: The data that support the findings of this study are available from the SRTR (https://www.srtr.org/requesting-srtr-data/data-requests/), Eurotransplant (https://www.eurotransplant.org/contact/data-and-study-requests/), and Scandiatransplant (http://www.scandiatransplant.org). However, restrictions apply to the availability of these data, which were used under license for the current study and are not publicly available. Data are available from the corresponding author upon reasonable request and with the explicit permission of the respective registries. Requests to access these datasets should be directed to Dongmei Yang, Yangdm1983@126.com.

## References

[1] Lund LH Khush KK Cherikh WS Goldfarb S Kucheryavaya AY Levvey BJ The registry of the international society for heart and lung transplantation: thirty-fourth adult heart transplantation report-2017; focus theme: allograft ischemic time. J Heart Lung Transplant. (2017) 36:1037–46. 10.1016/j.healun.2017.07.019 28779893

[2] Smits JM. Actual situation in eurotransplant regarding high urgent heart transplantation. Eur J Cardiothorac Surg. (2012) 42:609–11. 10.1093/ejcts/ezs424 22869253

[3] Colvin M Smith JM Ahn Y Skeans MA Messick E Goff R OPTN/SRTR 2019 annual data report: heart. Am J Transplant. (2021) 21(Suppl 2):356–440. 10.1111/ajt.16492 33595196

[4] Martin CM Moller JH. Congestive Heart Failure and Cardiac Transplantation: Clinical, Pathology, Imaging and Molecular Profiles. Cham: Springer International Publishing) (2017). p. 539–47.

[5] Pocock SJ Ariti CA McMurray JJ Maggioni A Kober L Squire IB Predicting survival in heart failure: a risk score based on 39 372 patients from 30 studies. Eur Heart J. (2013) 34:1404–13. 10.1093/eurheartj/ehs337 23095984

[6] Esteva A Robicquet A Ramsundar B Kuleshov V DePristo M Chou K A guide to deep learning in healthcare. Nat Med. (2019) 25:24–9. 10.1038/s41591-018-0316-z 30617335

[7] Chiu KC Du D Nair N Du Y 43rd Annual International Conference of the IEEE Engineering in Medicine & Biology Society (EMBC),1-5 Nov. 2021 2021), vol. Series). San Francisco, California, USA: Association for Computing Machinery (2021). pp 2144–7. 10.1109/EMBC46164.2021.9630234 34891713

[8] Rudin C. Stop explaining black box machine learning models for high stakes decisions and use interpretable models instead. Nat Mach Intell. (2019) 1:206–15. 10.1038/s42256-019-0048-x 35603010 PMC9122117

[9] Ribeiro MT Singh S Guestrin C. “Why should I trust you?”: explaining the predictions of any classifier. Proceedings of the 22nd ACM SIGKDD International Conference on Knowledge Discovery and Data Mining (2016). p. 1135–44

[10] Caruana R Lou Y Gehrke J Koch P Sturm M Elhadad N. Intelligible models for HealthCare: predicting pneumonia risk and hospital 30-day readmission. Proceedings of the 21th ACM SIGKDD International Conference on Knowledge Discovery and Data Mining, Sydney, NSW, Australia: Association for Computing Machinery (2015). p. 1721–30

[11] Little RJ Rubin DB. Statistical Analysis with Missing Data, Third Edition. Hoboken, NJ: John Wiley & Sons (2019).

[12] James G Witten D Hastie T Tibshirani R. An Introduction to Statistical Learning: with Applications in R. New York, NY: Springer (2013). 10.1007/978-1-4614-7138-7

[13] Moreno-Torres JG Raeder T Alaiz-Rodríguez R Chawla NV Herrera F. A unifying view on dataset shift in classification. Pattern Recogn. (2012) 45:521–30. 10.1016/j.patcog.2011.06.019

[14] Chawla NV Bowyer KW Hall LO Kegelmeyer WP. SMOTE: synthetic minority over-sampling technique. J. Artif. Int. Res. (2002) 16:321–57. 10.1613/jair.953

[15] Steyerberg EW. Clinical Prediction Models: A Practical Approach to Development, Validation, and Updating. New York, NY: Springer (2009). 10.1007/978-0-387-77244-8

[16] Nilsson J Ohlsson M Höglund P Ekmehag B Koul B Andersson B. The international heart transplant survival algorithm (IHTSA): a new model to improve organ sharing and survival. PLoS One. (2015) 10:e0118644. 10.1371/journal.pone.0118644 25760647 PMC4356583

[17] United Network for Organ Sharing (UNOS). UNOS/OPTN Heart Transplant Data 1987–2024. (2024). Available online at: https://optn.transplant.hrsa.gov/data/ (Accessed March 15, 2025).

[18] Dellgren G Geiran O Lemström K Gustafsson F Eiskjaer H Koul B Three decades of heart transplantation in scandinavia: long-term follow-up. Eur J Heart Fail. (2013) 15:308–15. 10.1093/eurjhf/hfs160 23109651

[19] Aleksova N Alba AC Molinero VM Connolly K Orchanian-Cheff A Badiwala M Risk prediction models for survival after heart transplantation: a systematic review. Am J Transplant. (2020) 20:1137–51. 10.1111/ajt.15708 31733026

[20] Medved D Ohlsson M Höglund P Andersson B Nugues P Nilsson J. Improving prediction of heart transplantation outcome using deep learning techniques. Sci Rep. (2018) 8:3613. 10.1038/s41598-018-21417-7 29483521 PMC5827028

[21] Van Calster B McLernon DJ van Smeden M Wynants L Steyerberg EW; Topic Group ‘Evaluating diagnostic tests and prediction models’ of the STRATOS initiative. Calibration: the achilles heel of predictive analytics. BMC Med. (2019) 17:230. 10.1186/s12916-019-1466-7 31842878 PMC6912996

[22] Kolsrud O Karason K Holmberg E Ricksten SE Felldin M Samuelsson O Renal function and outcome after heart transplantation. J Thorac Cardiovasc Surg. (2018) 155:1593–604 e1. 10.1016/j.jtcvs.2017.11.087 29338859

[23] Jernryd V Stehlik J Metzsch C Lund LH Gustav Smith J Andersson B Donor age and ischemic time in heart transplantation—implications for organ preservation. J Heart Lung Transplant. (2025) 44:364–75. 10.1016/j.healun.2024.10.030 39491603

